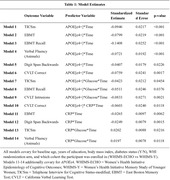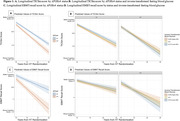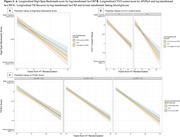# *APOEε*4, blood glucose levels, peripheral inflammation, and cognitive change among postmenopausal women in the Women’s Health Initiative

**DOI:** 10.1002/alz.094586

**Published:** 2025-01-09

**Authors:** Hannah M Klinger, Gillian T Coughlan, Kathleen M. Hayden, JoAnn E. Manson, Robert A Wild, Su Yon Jung, Ramon Casanova, Longjian Liu, Aladdin H. Shadyab, Jean Wactawski‐Wende, Susan M Schembre, Simin Liu, Stephen R. Rapp, Susan M. Resnick, Suzanne L. Baker, Rachel F Buckley

**Affiliations:** ^1^ Massachusetts General Hospital, Boston, MA USA; ^2^ Massachusetts General Hospital, Harvard Medical School, Boston, MA USA; ^3^ Wake Forest University School of Medicine, Winston Salem, NC USA; ^4^ Brigham and Women’s Hospital, Harvard Medical School, Boston, MA USA; ^5^ The University of Oklahoma Health Sciences, Oklahoma City, OK USA; ^6^ University of California, School of Nursing; Department of Epidemiology, Fielding School of Public Health, Los Angeles, CA USA; ^7^ Wake Forest University School of Medicine, Winston‐Salem, NC USA; ^8^ Drexel University Dornsife School of Public Health, Philadelphia, PA USA; ^9^ Herbert Wertheim School of Public Health and Human Longevity Science, University of California, San Diego, La Jolla, CA USA; ^10^ University at Buffalo School of Public Health and Health Professions, Buffalo, NY USA; ^11^ Georgetown’s Lombardi Comprehensive Cancer Center, Washington, DC USA; ^12^ Brown University School of Public Health, Boston, MA USA; ^13^ National Institute on Aging, National Institutes of Health, Baltimore, MD USA; ^14^ Lawrence Berkeley National Laboratory, Berkeley, CA USA

## Abstract

**Background:**

Older women have a higher prevalence of Alzheimer’s disease relative to comparably aged men. Although the biological mechanisms driving this sex difference remain elusive, emerging data suggest that longevity, *APOEε*4, inflammation, and blood glucose levels may play roles. Our objective was to examine the associations of *APOEε*4, high sensitivity‐C‐reactive protein (hs‐CRP), and fasting blood glucose levels with cognitive changes over time among women enrolled in two ancillary studies of the Women’s Health Initiative (WHI).

**Method:**

We identified 2,534 women from WHIMS‐ECHO (Enrollment age_mean(SD)_ = 70(4) years) and 400 women from WHIMS‐Y (Enrollment age_mean(SD)_ = 52(1) years) with baseline data available for *APOEε*4 carriage, plasma hs‐CRP, and serum‐measured fasting blood glucose, and longitudinal cognitive measures, including the TICSm, EBMT, EBMT‐recall, verbal fluency, Digit Span Backwards, and CVLT scores. A series of linear mixed‐effects models were used to examine these three predictors’ main and interactive effects on longitudinal cognition changes, adjusting for age, education level, body mass index, diabetes status, WHI randomization arm, and cohort (including random intercepts and slopes). Hs‐CRP levels were log transformed and glucose levels were inverse transformed. All data were z‐scored.

**Result:**

*APOEε*4 carriage was associated with steeper cognitive decline on the TICSm, EMBT, EBMT‐recall, verbal fluency, Digit Span Backwards, and CVLT‐correct scores compared to non‐carriers (Table 1). *APOEε*4 carriers with lower blood glucose levels showed steeper cognitive decline on TICSm and EBMT‐recall scores but less intrusions on the CVLT compared to *APOEε*4 carriers with higher glucose levels. Additionally, *APOEε*4 carriers with higher levels of hs‐CRP demonstrated a steeper decline on CVLT‐correct scores relative to *APOEε*4 carriers with lower hs‐CRP levels. Women with higher levels of hs‐CRP alone exhibited steeper cognitive decline on the EBMT and Digit Span Backwards test compared to women with lower hs‐CRP. Women with higher levels of both hs‐CRP and blood glucose also showed significantly faster rates of decline on TICSm and verbal fluency scores relative to women with higher hs‐CRP who had lower blood glucose levels. Three‐way interaction tests (*APOEε*4*CRP*glucose) did not yield significant results.

**Conclusion:**

These findings underscore the complex interplay between genetic risk, metabolic and inflammatory health, and their associations with cognitive decline among older women.